# 5-Aminolevulinic Acid Protects against Cisplatin-Induced Nephrotoxicity without Compromising the Anticancer Efficiency of Cisplatin in Rats In Vitro and In Vivo

**DOI:** 10.1371/journal.pone.0080850

**Published:** 2013-12-06

**Authors:** Yoshio Terada, Keiji Inoue, Tatsuki Matsumoto, Masayuki Ishihara, Kazu Hamada, Yoshiko Shimamura, Koji Ogata, Kosuke Inoue, Yoshinori Taniguchi, Taro Horino, Takashi Karashima, Kenji Tamura, Hideo Fukuhara, Shimpei Fujimoto, Masayuki Tsuda, Taro Shuin

**Affiliations:** 1 Department of Endocrinology, Metabolism and Nephrology, Kochi Medical School, Kochi University, Kohasu, Oko-cho, Nankoku, Japan; 2 Department of Urology, Kochi Medical School, Kochi University, Kohasu, Oko-cho, Nankoku, Japan; 3 Institute for Laboratory Animal Research, Kochi Medical School, Kochi University, Kohasu, Oko-cho, Nankoku, Japan; Emory University, United States of America

## Abstract

**Background/Aims:**

Nephrotoxicity is a frequent and major limitation in cisplatin (CDDP)-based chemotherapy. 5-Aminolevulinic acid (ALA) is widely distributed in animal cells, and it is a precursor of tetrapyrole compounds such as heme that is fundamentally important in aerobic energy metabolism. The aim of this study is to evaluate the protective role of ALA in CDDP-induced acute kidney injury (AKI).

**Method:**

We used CDDP-induced AKI rat model and cultured renal tubular cells (NRK-52E). We divided four groups of rats: control, CDDP only, CDDP + ALA(post);(ALA 10 mg/kg + Fe in drinking water) after CDDP, CDDP + ALA(pre & post).

**Result:**

CDDP increased Cr up to 6.5 mg/dl, BUN up to 230 mg/dl, and ALA significantly reduced these changes. ALA ameliorates CDDP-induced morphological renal damages, and reduced tubular apoptosis evaluated by TUNEL staining and cleaved caspase 3. Protein and mRNA levels of ATP5α, complex(COX) IV, UCP2, PGC-1α in renal tissue were significantly decreased by CDDP, and ALA ameliorates reduction of these enzymes. In contrast, Heme Oxigenase (HO)-1 level is induced by CDDP treatment, and ALA treatment further up-regulates HO-1 levels. In NRK-52E cells, the CDDP-induced reduction of protein and mRNA levels of mitochondrial enzymes was significantly recovered by ALA + Fe. CDDP-induced apoptosis were ameliorated by ALA + Fe treatment. Furthermore, we evaluated the size of transplantated bladder carcinoma to the rat skin, and ALA did not change the anti cancer effects of CDDP.

**Conclusion:**

These data suggested that the protective role of ALA in cisplatin-induced AKI is via protection of mitochondrial viability and prevents tubular apoptosis. Also there are no significant effects of ALA on anticancer efficiency of CDDP in rats. Thus, ALA has the potential to prevent CDDP nephrotoxicity without compromising its anticancer efficacy.

## Introduction

Cisplatin is one of the most effective and potent anticancer drugs in the treatment of epithelial malignancies such as lung, head and neck, ovarian, bladder, and testicular cancers [1]. The major constraint to cisplatin-based chemotherapy is the frequent development of nephrotoxicity [2]. The antineoplastic effect of cisplatin is dose dependent, yet the risk of nephrotoxicity often precludes the use of higher doses to maximize the therapeutic effect. Cisplatin induces apoptosis of renal proximal tubule cells (LLC-PK1) in vitro by means of mitochondria-dependent and -independent pathways [3], partly through the activation of caspase-3 [4]. Oxidant stress also appears to contribute to the cisplatin-induced apoptosis of renal tubular cells, both in vitro and in vivo [5]. Several studies, including ours, suggest that caspase inhibitors and knockout of apoptosis-related genes attenuate cisplatin-induced acute kidney injury (AKI) in rats [6], [7]. Mitochondria have a variety of important intracellular functions, including ATP production, synthesis of reactive oxygen species, and regulation of the cell death pathway. Recent studies, including ours, have demonstrated that mitochondrial function is one of the key factors protecting cells from oxidative stress in AKI [8], [9]. Changes in mitochondrial structure and membrane potential were reported in the proximal tubules during AKI [8], [9].

5-Aminolevulinic acid (ALA) is the naturally occurring metabolic precursor of an endogenously synthesized photosensitizer, protoporphyrin IX (PpIX) [10]–[12]. ALA is widely distributed in animal cells, and it is a precursor of tetrapyrole compounds such as heme, which is fundamentally important in aerobic energy metabolism [13]. Here, we explored the relevance of ALA in protecting renal tubular cells in cisplatin-treated rats through the attenuation of mitochondrial enzymes and the apoptotic pathway. Thus, ALA has the potential to prevent cisplatin nephrotoxicity without compromising the anticancer efficacy of cisplatin. This study was conducted to determine whether ALA affects the course of cisplatin-induced AKI. To achieve this, we examined differences in the renal function, histology, changes of mitochondrial enzymes, and tubular cell apoptosis in cisplatin-induced AKI. Our data demonstrated that ALA has the potential to prevent cisplatin nephrotoxicity without compromising the anticancer efficacy of cisplatin.

## Materials and Methods

### Induction of cisplatin-induced AKI

Male Sprague-Dawley rats (Saitama Experimental Animal Supply, Saitama, Japan) weighing 150–200 g were anesthetized by intraperitoneal injection with sodium pentobarbital (30 mg/kg). Cisplatin (Sigma-Aldrich, St. Louis, MO, USA) was dissolved in saline at a concentration of 1 mg/mL. The rats were given a single intraperitoneal injection of either a vehicle (saline) or cisplatin (8 mg/kg body weight). 5-ALA 10 mg/kg + Fe (sodium ferrous citrate, 15.7 mg/kg) dissolved in drinking water were administered to rats. 5-ALA (COSMO BIO co., ltd. Tokyo, Japan), and sodium ferrous citrate (kindly provided by SBI Pharmaceuticals Co., Ltd., Tokyo, Japan) were prepared. 5-ALA and Fe were purchased form Sigma-Aldrich (St. Louis, MO, USA). The animals were divided into 4 subgroups: (1) a control (saline) group, (2) a cisplatin group, (3) an ALA-treated post-cisplatin-injection group (post), and (4) an ALA-treated pre- and post-cisplatin-injection group (pre & post) (n = 8 for each group). The blood was obtained via tail vein at 1, 3, 5, 7, and 9 days after cisplatin injection. The rats were killed at 5 and 9 days after surgery (Figure S1). The left kidney was rapidly removed and processed for histological evaluation, protein extraction, and RNA extraction at day 5 and 9 as previously described [14], [15]. All animal protocols were approved by the Institutional Animal Care and Use Committee at the University of Kochi (#20–027), and experiments were conducted in accordance with institutional guidelines. All surgery was performed under sodium pentobarbital anesthesia, and all efforts were made to minimize suffering.

### Effects of ALA on the antitumorigenic effects of cisplatin

Male F344/NJcl-rnu/rnu rats (immunodeficiency rats) (Saitama Experimental Animal Supply) weighing 150–200 g were anesthetized by intraperitoneal injection with sodium pentobarbital (30 mg/kg). 253J-BV (a bladder carcinoma cell line) cells (2×10^7^) originally purchased from American Type Culture Collection (Manassas, VA, USA) were subcutaneously injected into the skin of the back. The rats were given a single intraperitoneal injection of either a vehicle (saline) or cisplatin (8 mg/kg body weight). 5-ALA 10 mg/kg + Fe (sodium ferrous citrate, 15.7 mg/kg) dissolved in drinking water were administered to rats. The animals were divided into 4 subgroups: (1) a control (saline) group, (2) a cisplatin group, (3) an ALA-treated post-cisplatin-injection group (post), and (4) an ALA-treated pre- and post-cisplatin-injection group (pre & post) (n = 5 for each group). The volume of the carcinoma was measured at 1, 3, 5, 7, and 9 days after surgery.

### Cell culture

NRK-52E cells (renal tubular cells from adult rats), originally purchased from American Type Culture Collection, were grown in Dulbecco's modified Eagle's medium (Gibco, Rockville, MD, USA) supplemented with 50 IU/mL penicillin and 10% heat-inactivated fetal calf serum (Gibco) [15]. For the cisplatin experiments, cisplatin (20 μM) was added to the NRK-52E cells for 24 h. For the ALA + cisplatin experiments, 200 μM 5-ALA, 100 μM sodium ferrous citrate, and 20 μM cisplatin were added to the NRK-52E cells for 24 h. All other chemicals were purchased from Funakoshi (Tokyo, Japan).

### Isolation and histological examination of kidney tissue

Rats were anesthetized with pentobarbital at the indicated times after cisplatin administration. The kidneys were perfused in situ with sterile phosphate-buffered saline (PBS) and the left kidney was then rapidly excised, frozen in liquid nitrogen, and homogenized in SDS sample buffer, as described previously [16]. For immunohistochemical studies, kidneys were fixed in formalin overnight, dehydrated, and embedded in paraffin. Thin sections were cut and subjected to periodic acid-Schiff staining, as described previously. Tubular injury was assessed by using a semiquantitative scale [6]. Histological changes due to tubular necrosis were quantitated by determining the percentage of tubules with evident cell necrosis, loss of brush border, cast formation, and tubule dilatation, as follows: 0 =  none, 1 = ≤10%, 2 = 11%–25%, 3 = 26%–45%, 4 = 46%–75%, and 5 = ≤76%. At least 5–10 fields (×200) were reviewed for each slide. The Apoptosis TUNEL Kit II (MBL, Tokyo, Japan) was used for the staining of terminal deoxynucleotidyl transferase-mediated dUTP nick end labeling (TUNEL)-positive cells, as previously described [6].

### Western blot analysis

Protein extracts of the total renal tissue or NRK-52E cells (50 μg samples) were prepared and denatured by heating at 100°C for 5 min in SDS sample buffer as described previously [17]. The proteins were separated on 7.5% or 10%–20% polyacrylamide gels and transferred to nitrocellulose membranes. The membranes were blocked for 1 h with 5% (wt/vol) fat-free milk in PBS and probed with the appropriate primary antibodies (anti-ATP5α, anti-complex [COX]-IV, anti-PGC-1α, anti-UCP2, anti-nitrotyrosine [anti-NT], anti-procaspase-3, or anti-actin [Santa Cruz Biochemicals Inc., Santa Cruz, CA, USA]). The primary antibodies were detected with horseradish peroxidase (HRP)-conjugated rabbit anti-goat IgG or HRP-donkey anti-rabbit IgG, and visualized by using the Amersham ECL system (Amersham Corp., Arlington Heights, IL, USA).

### Measurements of heme

We measured heme to evaluate the metabolic product of ALA using assay kit (BioChain Institute, Newark, CA). Heme Assay Kit is based on an improved aqueous alkaline solution method, in which the heme is converted into a uniform colored form. The intensity of color, measured at 400 nm, is directly proportional to the heme concentration in the sample. We measured the renal tissue extract in the in vivo experiments, and the cell extracts in the in vivo experiments. The protein extracts of the total renal tissue or NRK-52E cells (100 μg samples) were prepared following the same method used in the immunoblot analysis.

### Real-time quantitative polymerase chain reaction

Reverse transcription-polymerase chain reaction (RT-PCR) analysis of RNA extracted from kidneys was carried out as previously described [16]. In brief, total RNA was isolated from renal tissues by using TRI Reagent (Life Technologies, Gaithersburg, MD, USA). Samples of total RNA (1 μg) were reverse transcribed, and real-time qPCR was performed to quantify changes in *ATP5*α, *COX-IV*, *PGC-1*α, and *UCP2* gene expression by using the ABI LightCycler real-time PCR system (ABI, Los Angeles, CA, USA). RT-PCR of glyceraldehyde-3-phosphate dehydrogenase (GAPDH) served as a positive control. A 3-step PCR was performed for 35 cycles. The samples were denatured at 94°C for 30 s, annealed at 58°C for 30 s, and extended at 72°C for 30 s. The primers were obtained from ABI.

### Mitochondrial morphology obtained by using laser confocal immunofluorescence microscopy

NRK-52E cells were stained with MitoTracker mitochondrion-selective probes (Invitrogen, Rockville, MD, USA). For confocal microscopy, NRK-52E cells were then fixed with 2% paraformaldehyde in PBS for 1 h and processed for imaging as described previously, and examined under a confocal laser microscope (Carl Zeiss Japan, Tokyo, Japan) [18]. Fragmented mitochondria were condensed and punctate, whereas normal mitochondria showed a threadlike or rounded structure. Cells with mitochondrial fragmentation were defined as those containing a majority (>70%) of fragmented mitochondria, and they were counted to determine the percentage in 50 cells/sample.

### Statistics

Results are presented as mean ± SEM. Differences between the groups were tested by 2-way analysis of variance (ANOVA) followed by Scheffe's test for multiple comparisons. Two groups were compared by using unpaired *t-*tests. A p value of <0.05 was considered statistically significant.

## Results

### 5-Aminolevulinic acid protects from cisplatin-induced renal injury

**Figure 1:**
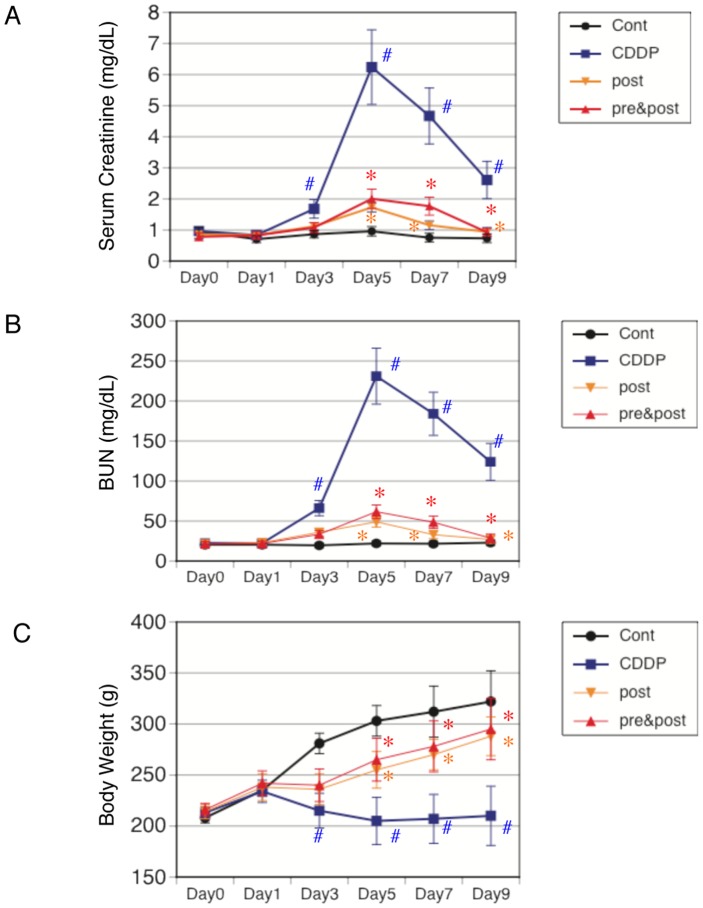
Blood urea nitrogen (BUN) and serum creatinine (Cre) levels in ALA treated rats after cisplatin injection. Rats were divided into four subgroups: 1) a control (saline) group, 2) a cisplatin group, 3) an ALA–treated post cisplatin-injection group, 4) an ALA–treated pre & post cisplatin-injection group (n = 8 for each group). Serum creatinine (A) and blood urea nitrogen (B), and body weight (C) were measured at the indicated times. Data are mean ± SEM of 8 rats per group. Statistically significant differences (*p<0.05 v.s. CDDP, #p<0.05 v.s. control) are indicated.

**Figure 2:**
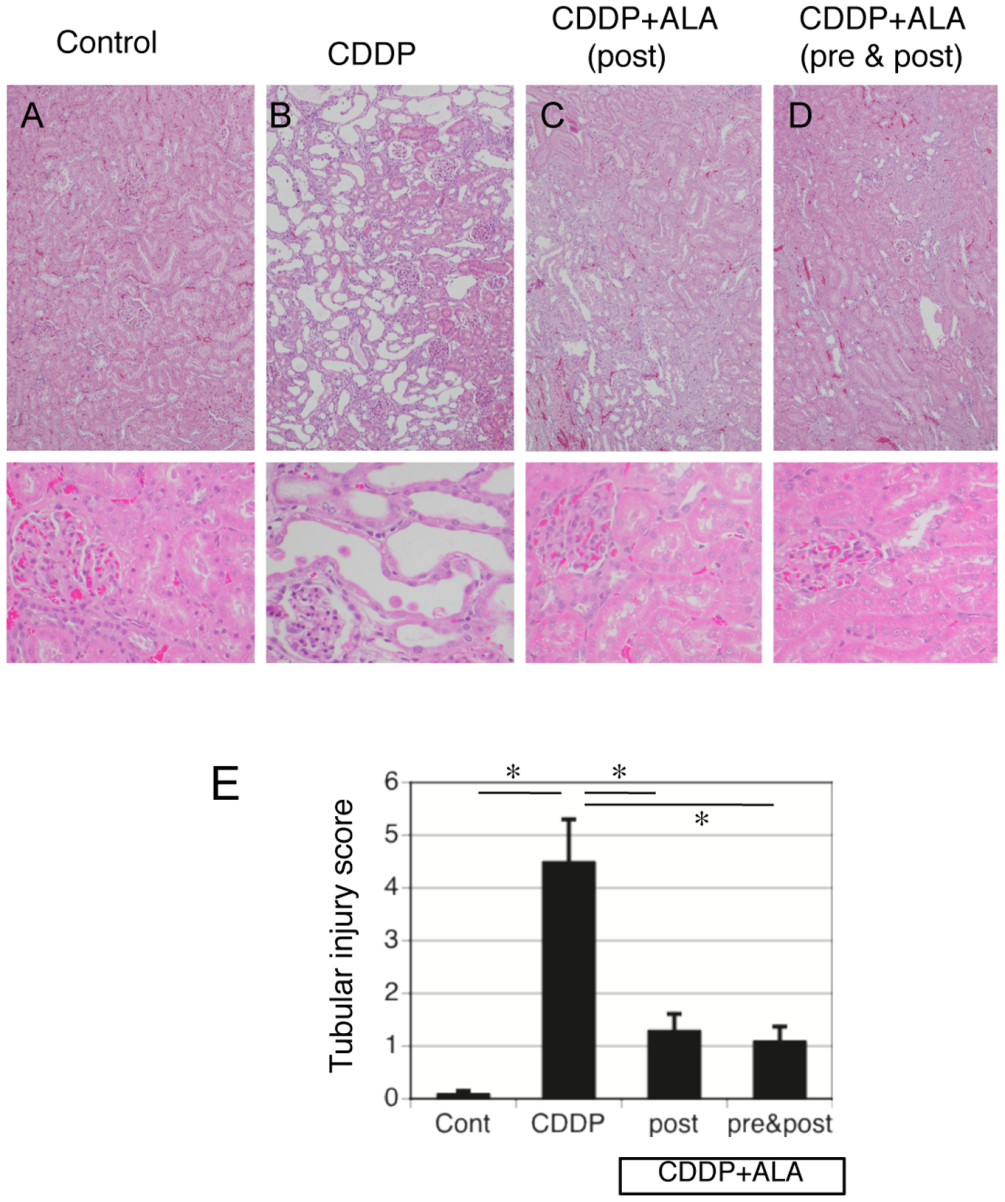
Renal histopathology and scores for characteristic histological signs of renal injury in ALA treated rats with cisplatin-induced AKI. A, B) Kidneys were removed 5 days after injection with cisplatin (8 mg/kg) or control. The cisplatin-treated rats exhibited acute structural damage characterized by tubular necrosis, swelling and tubular dilation, extensive epithelial vacuolization, and hyaline casts in renal tubules. C, D) Kidneys of ALA treatment (both post and pre & post) show a very slight loss of tubular epithelial cells and very low levels of intratubular debris and cast formation (Magnification, X50 upper, X200 lower figures) E) ALA reduced the renal injury score in cisplatin-treated rats. The semiquantitative histological injury score was significantly higher in cisplatin-treated rats than in controls. Data are the mean SEM of 6 rats per group. Statistically significant differences (* p<0.05) are indicated.

Cisplatin increased the serum blood urea nitrogen and creatinine levels in comparison with the controls at days 5–9. ALA treatments (both post and pre & post) significantly prevented these changes in cisplatin-treated animals (Figure 1A,B). Body weight was not significantly different in rats before the beginning of the treatment. Cisplatin reduced body weight gain in the test animals. ALA treatments (both post and pre & post) significantly prevented, but did not normalize, cisplatin-induced weight loss (Figure 1C). The toxic effect of cisplatin was also confirmed by the detection of morphologic abnormalities in kidney slices. The histology results for the control rats were normal (Figure 2A). The cisplatin group exhibited acute structural damage characterized by tubular necrosis, swelling and tubular dilation, extensive epithelial vacuolization, and hyaline casts in renal tubules (Figure 2B–D). ALA reduced these tubular damages in cisplatin-treated rats (Figure 2C,D). The semiquantitative histological injury score was significantly higher in cisplatin-treated rats than in controls (Figure 2E).

### 5-Aminolevulinic acid reduces cisplatin-induced apoptosis

**Figure 3:**
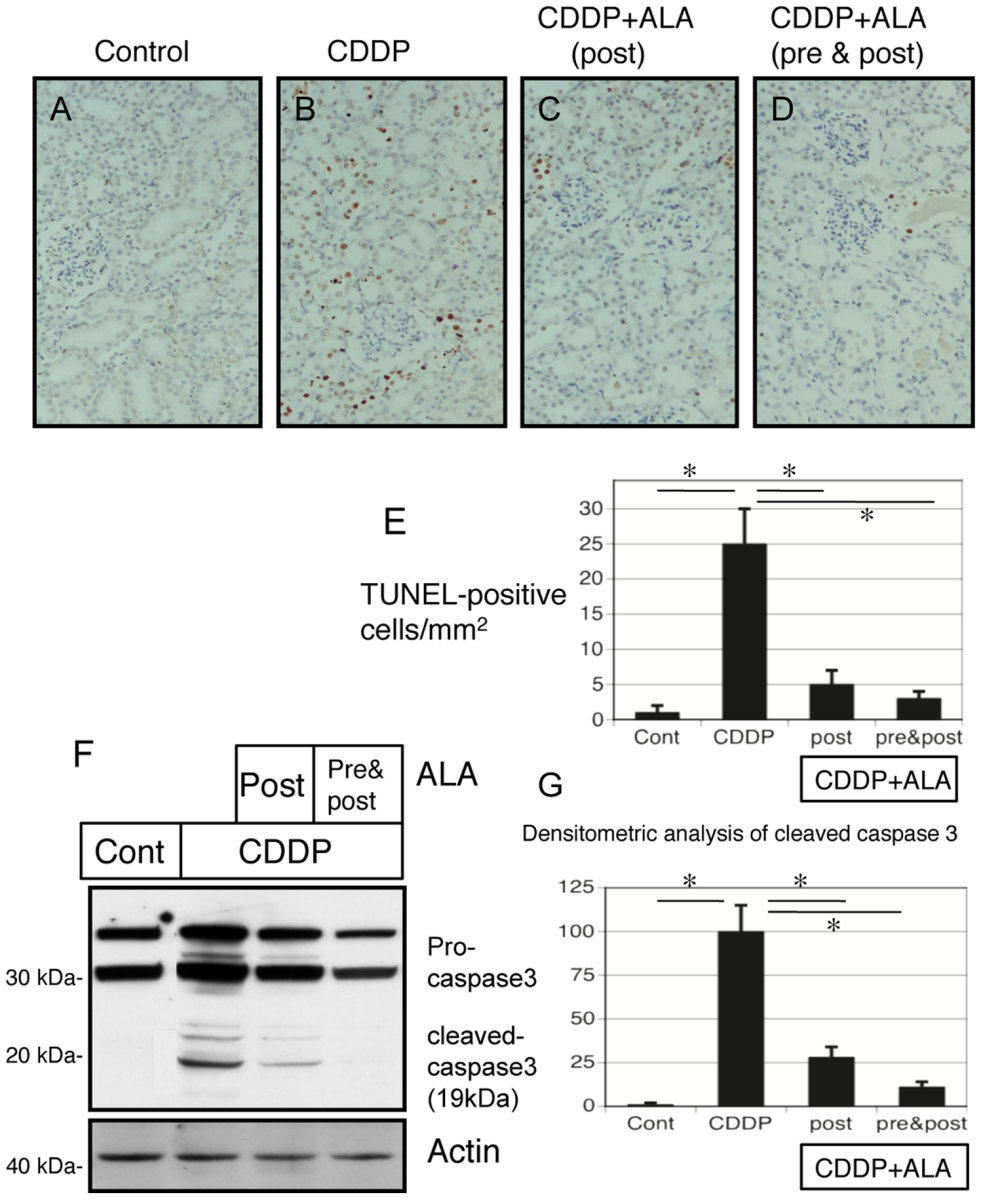
Tubular cell apoptosis and levels of cleaved caspase3 in renal tissues of ALA treated rats with cisplatin-inducedAKI. Kidneys were removed 5(8 mg/kg). A, B) Kidneys of cisplatin-treated rats exhibited an elevated number of TUNEL-positive renal tubular cells. (Magnification, X100). C, D) Kidneys of ALA treatment (both post and pre & post) show a very few number of TUNEL-positive renal tubular cells. (Magnification, X100) E) The number of TUNEL positive tubular cells was significantly low in ALA (both post and pre & post) treated rat kidneys in cisplatin-induced AKI. (F) Western blot analysis of cleaved caspase3 were performed in each group rats. (G) Quantitative densitometry was performed for cleaved caspase 3 blots. Data are the mean ± SEM of 6 rats per group. Statistically significant differences (*p<0.05) are indicated.

Apoptosis in the kidney was assessed by using the TUNEL assay. Cisplatin increased the number of apoptotic nuclei compared with the control group (Figure 3A,B). ALA treatments (both post and pre & post) significantly decreased the number of TUNEL-positive cells (Figure 3C,D). The number of TUNEL-positive cells were increased by cisplatin treatment and reduced by ALA administration (both post and pre & post) on quantitative analysis (Figure 3E). Cleaved caspase-3 levels were high in the renal tissue of rats treated with cisplatin. ALA treatments (both post and pre & post) significantly lowered the elevated caspase-3 levels in cisplatin-injected rat kidneys, as assessed by western blotting of the renal tissue and densitometric analysis (Figure 3F,G).

### 5-Aminolevulinic acid ameliorates cisplatin-induced reduction of mRNA and protein expression of mitochondria-related genes in vivo

**Figure 4:**
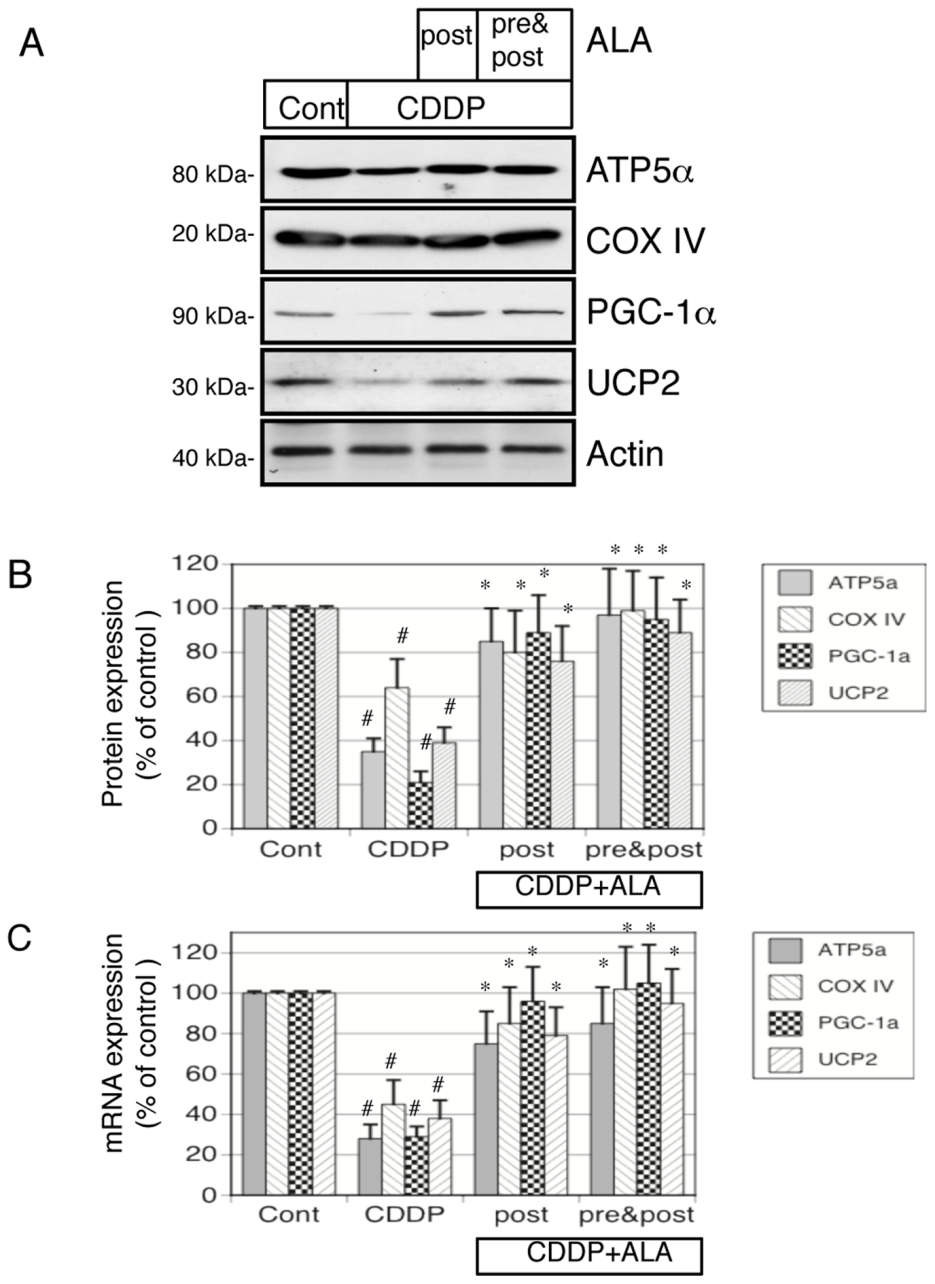
Western blot analyses of protein expression and RT-PCR analysis of mitochondria-related gene expression in ALA treated cisplatin-induced AKI rats. (A) Aliquots of 50 μg of protein from renal tissue extracts were separated by SDS-PAGE and transferred to membranes. Western blots analyses were performed for ATP5α, complex (COX)-IV, PGC-1α, UCP2 in cisplatin-treated, control, and cisplatin + ALA (both post and pre & post) treated rats. Actin served as a loading control. (B) Quantitative densitometry was performed for ATP5α, complex (COX)-IV, PGC-1α, and UCP2 western blots. (C) Quantitative analysis of mRNA was performed using RT-PCR for ATP5α, complex (COX)-IV, PGC-1α, and UCP2. GAPDH served as a loading control. Bars represent the mean ± SEM, n = 6. *p<0.05 v.s. CDDP, #p<0.05 v.s. control by ANOVA.

We next examined whether ALA protects the mitochondrial enzymes from cisplatin injury. Because previous studies demonstrated that cisplatin caused a marked decrease in the expression of several mitochondrial enzymes, we examined typical enzymes (ATP5α, COX-IV, PGC-1α, and UCP2) in cisplatin-treated, control, and cisplatin + ALA–treated rats. As shown in Figure 4A, cisplatin induced a significant loss of ATP5α, COX-IV, PGC-1α, and UCP2 protein expression. ALA treatment (both post and pre & post) recovered the cisplatin-induced decreases of ATP5α, COX-IV, PGC-1α, and UCP2 protein expression. Quantitative analysis revealed that cisplatin induced decreases of ATP5α, COX-IV, PGC-1α, and UCP2 protein expression, and these reductions were ameliorated by ALA treatments (both pre and pre & post) (Figure 4B). Furthermore, to examine the changes in the expression of these enzymes during cisplatin-induced AKI, we conducted an RT-PCR analysis of rat renal tissue mRNA. The mRNA levels of ATP5α, COX-IV, PGC-1α, and UCP2 were dramatically decreased by cisplatin, and these reductions were ameliorated by ALA treatments (both pre and pre & post) (Figure 4C).

### 5-Aminolevulinic acid protects against cisplatin-induced oxidative stress and induces heme oxygenase-1 expression in vivo

**Figure 5:**
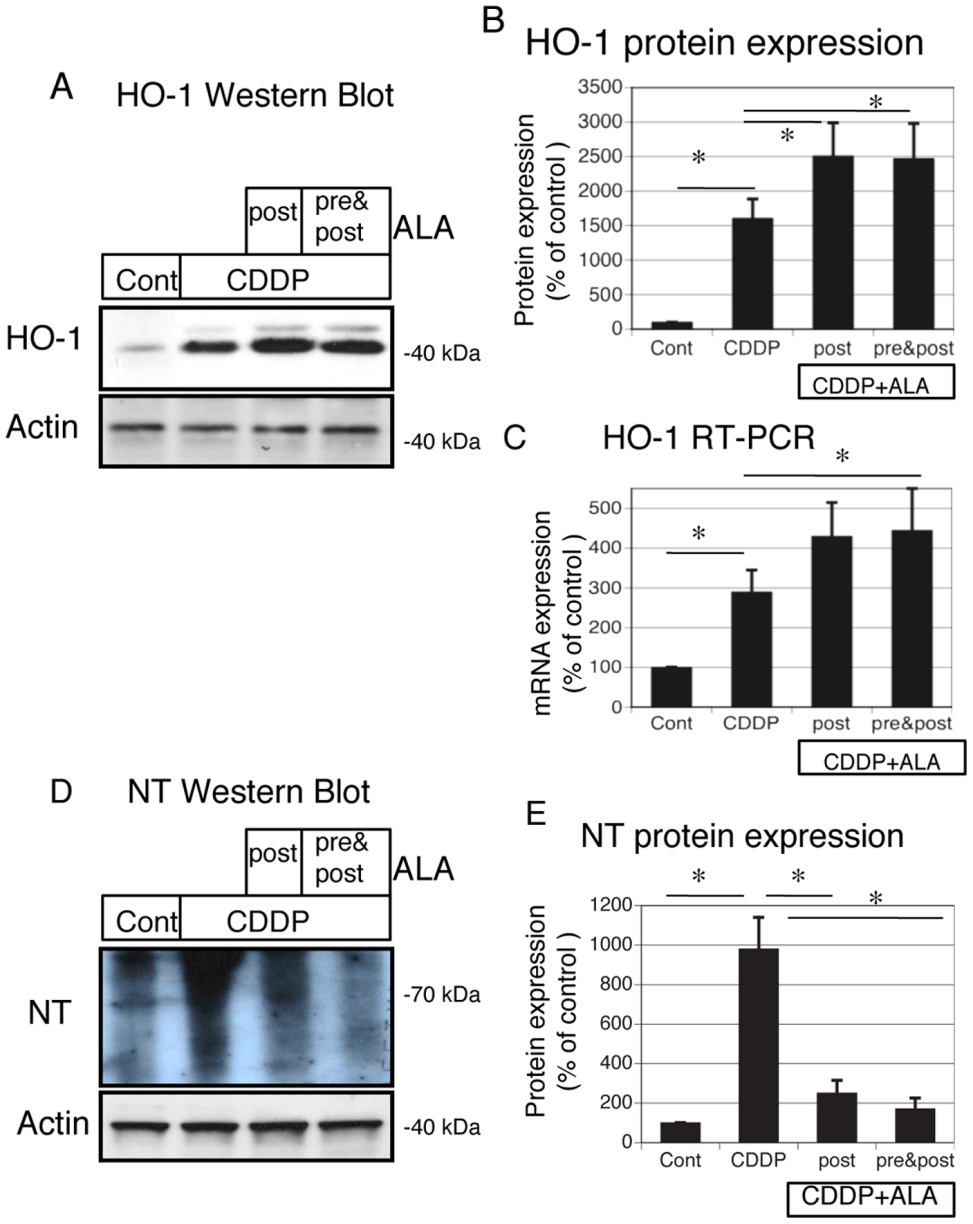
Protective effects of ALA to cisplatin-induced oxidative stress and induction of Heme oxygenase (HO)-1 expression in vivo. (A, D) Aliquots of 50 μg of protein from renal tissue extracts were separated by SDS-PAGE and transferred to membranes. Western blots analyses were performed for HO-1 (A), nitrotyrosine (NT) (D) in cisplatin-treated, control, and cisplatin + ALA (both post and pre & post) treated rats. Actin served as a loading control. (B) Quantitative densitometry was performed for HO-1 western blots. (C) Quantitative analysis of mRNA was performed using RT-PCR for HO-1. GAPDH served as a loading control (E) Quantitative densitometry was performed for NT western blots. Bars represent the mean ± SEM, n = 6. *P<0.05 by ANOVA.

We next examined the renal HO-1 expression in the 4 groups of rats. HO-1 mRNA and protein expression was induced by cisplatin treatment in vivo (Figure 5A–C). ALA treatments (both post and pre & post) further increased HO-1 expression (Figure 5A–C). Oxidative stress is a major factor causing renal injury in response to cisplatin. We examined the level of oxidative stress by using a typical marker, NT. On immunoblotting, NT was highly expressed in the cisplatin-treated kidney compared with the control (Figure 5D,E). However, treatments with ALA (both post and pre & post) reduced these signals (Figure 5D,E). These data suggest that cisplatin induced oxidative stress in damaged tubules, whereas such stress was significantly blocked by the ALA treatment.

### 5-Aminolevulinic acid and Fe protect against cisplatin-induced reduction of mRNA and protein expression of mitochondria-related genes in NRK-52E cells

**Figure 6:**
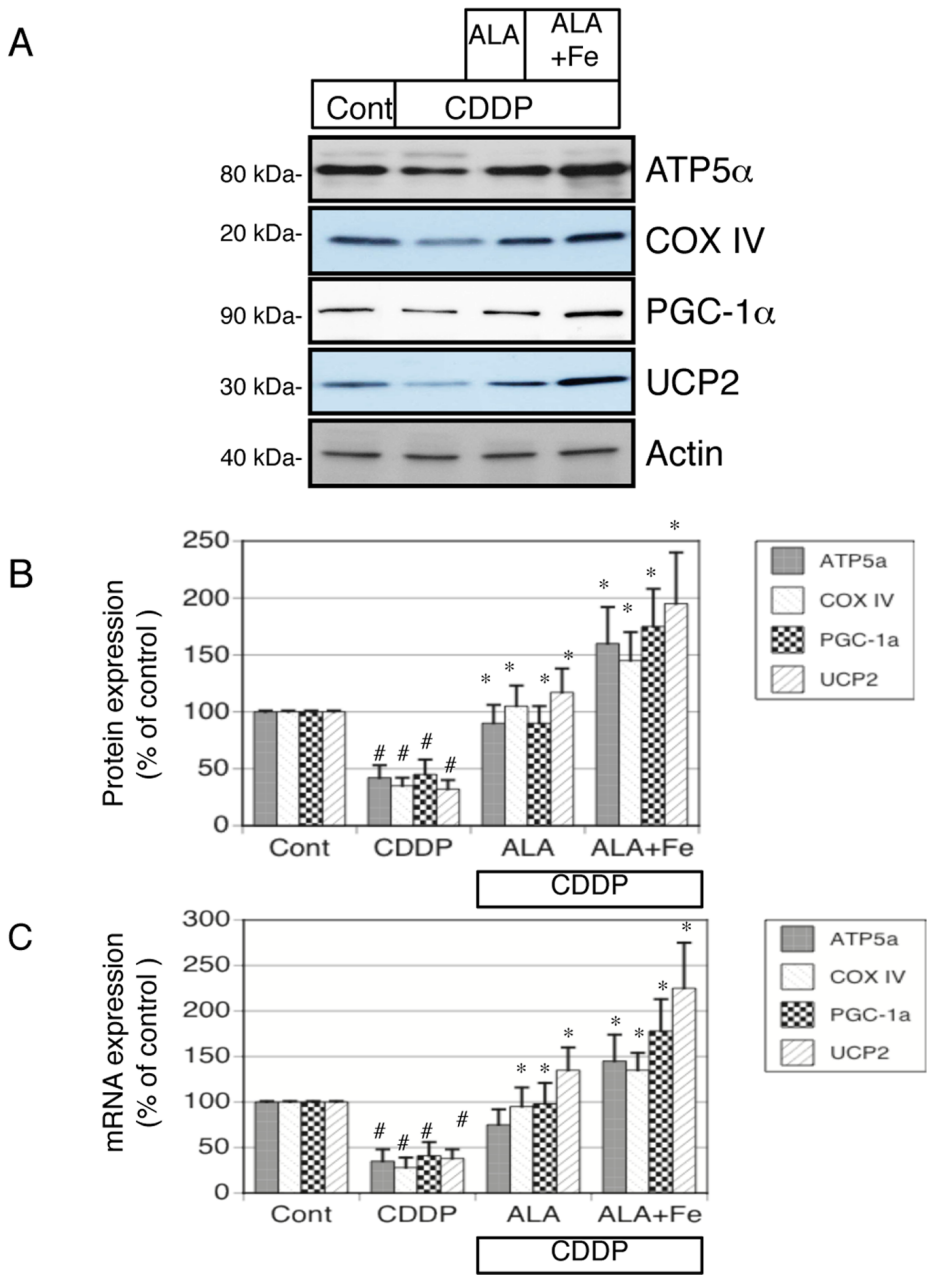
Western blot analyses of protein expression and RT-PCR analysis of mitochondria-related gene expression in ALA + Fe treated cisplatin-induced renal tubular injury. (A) Aliquots of 50 μg of protein extracts from NRK-52E cells were separated by SDS-PAGE and transferred to membranes. Western blots analyses were performed for ATP5α, complex (COX)-IV, PGC-1α, UCP2 in control, cisplatin, cisplatin + ALA, and cisplatin + ALA + Fe treated NRK-52E cells. Actin served as a loading control. (B) Quantitative densitometry was performed for ATP5α, complex (COX)-IV, PGC-1α, and UCP2 blots. (C) Quantitative analysis of mRNA was performed using RT-PCR for ATP5α, complex (COX)-IV, PGC-1α, and UCP2. Bars represent the mean ± SEM, n = 6. *p<0.05 v.s. CDDP, #p<0.05 v.s. control by ANOVA.

We next examined whether ALA and Fe protect the mitochondrial enzymes from cisplatin injury in vitro. We examined the typical enzymes (ATP5α, COX-IV, PGC-1α, and UCP2) in the control, cisplatin-, cisplatin + ALA-, and cisplatin + ALA + Fe–treated NRK-52E cells. As shown in Figure 6A,B, cisplatin induced a significant loss of ATP5α, COX-IV, PGC-1α, and UCP2 protein expression. ALA-, and ALA + Fe–treatment completely recovered the cisplatin-induced decreases in the expression of these proteins. Furthermore, to examine the changes in the mRNA expression of ATP5α, COX-IV, PGC-1α, and UCP2 after cisplatin treatment, we conducted RT-PCR analysis of mRNA in NRK-42E cells. The mRNA level of these enzymes was dramatically decreased by cisplatin, and these reductions were ameliorated by addition with ALA and, more effectively, ALA + Fe (Figure 6C).

### ALA and Fe protect against cisplatin-induced oxidative stress and induce HO-1 expression in NRK-52E cells

**Figure 7:**
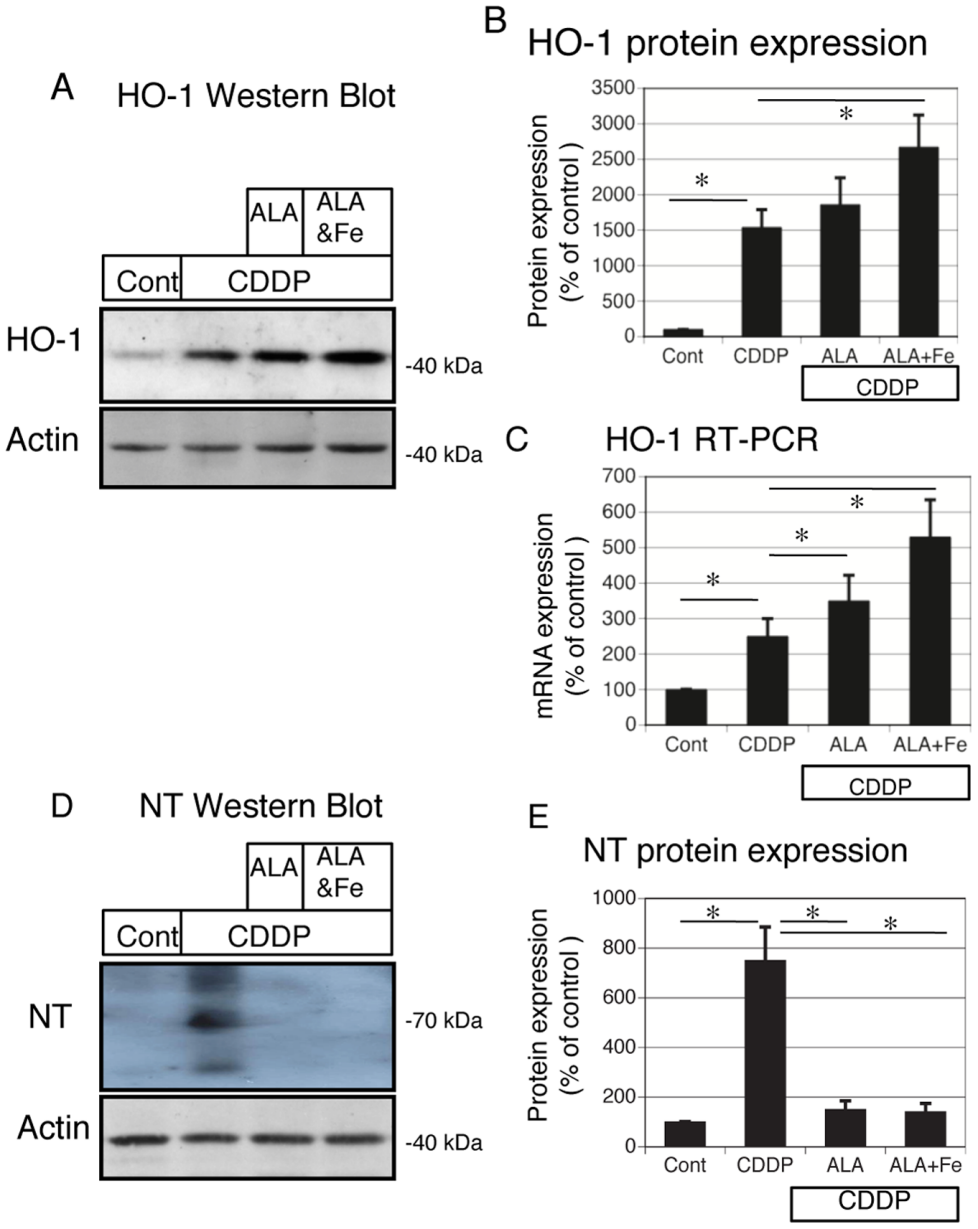
Protective effects of ALA + Fe to cisplatin-induced oxidative stress and induction of Heme oxygenase (HO)-1 expression in NRK-52E cells. (A, D) Aliquots of 50 μg of protein extracts from NRK-52E cells were separated by SDS-PAGE and transferred to membranes. Western blots analyses were performed for HO-1 (A), nitrotyrosine (NT) (D) in control, cisplatin, cisplatin + ALA, and cisplatin + ALA + Fe treated NRK-52E cells. Actin served as a loading control. (B) Quantitative densitometry was performed for HO-1 blots. (C) Quantitative analysis of mRNA was performed using RT-PCR for HO-1. (E) Quantitative densitometry was performed for NT blots. Bars represent the mean ± SEM, n = 6. *P<0.05 by ANOVA.

We next examined the HO-1 expression in the presence of cisplatin, ALA, and Fe. HO-1 mRNA and protein expression was induced by cisplatin treatment in NRK-52E cells. ALA treatment additionally increased HO-1 expression. ALA + Fe treatment significantly upregulated HO-1 expression in the presence of cisplatin (Figure 7A–C). Oxidative stress is a major factor causing renal injury in response to cisplatin. On immunoblotting, NT was highly induced by cisplatin treatment compared with the control. Treatment with ALA and Fe significantly reduced these signals (Figure 7D,E).

### ALA and Fe prevent cisplatin-induced damage of mitochondrial structure and apoptosis in NRK-52E cells

**Figure 8:**
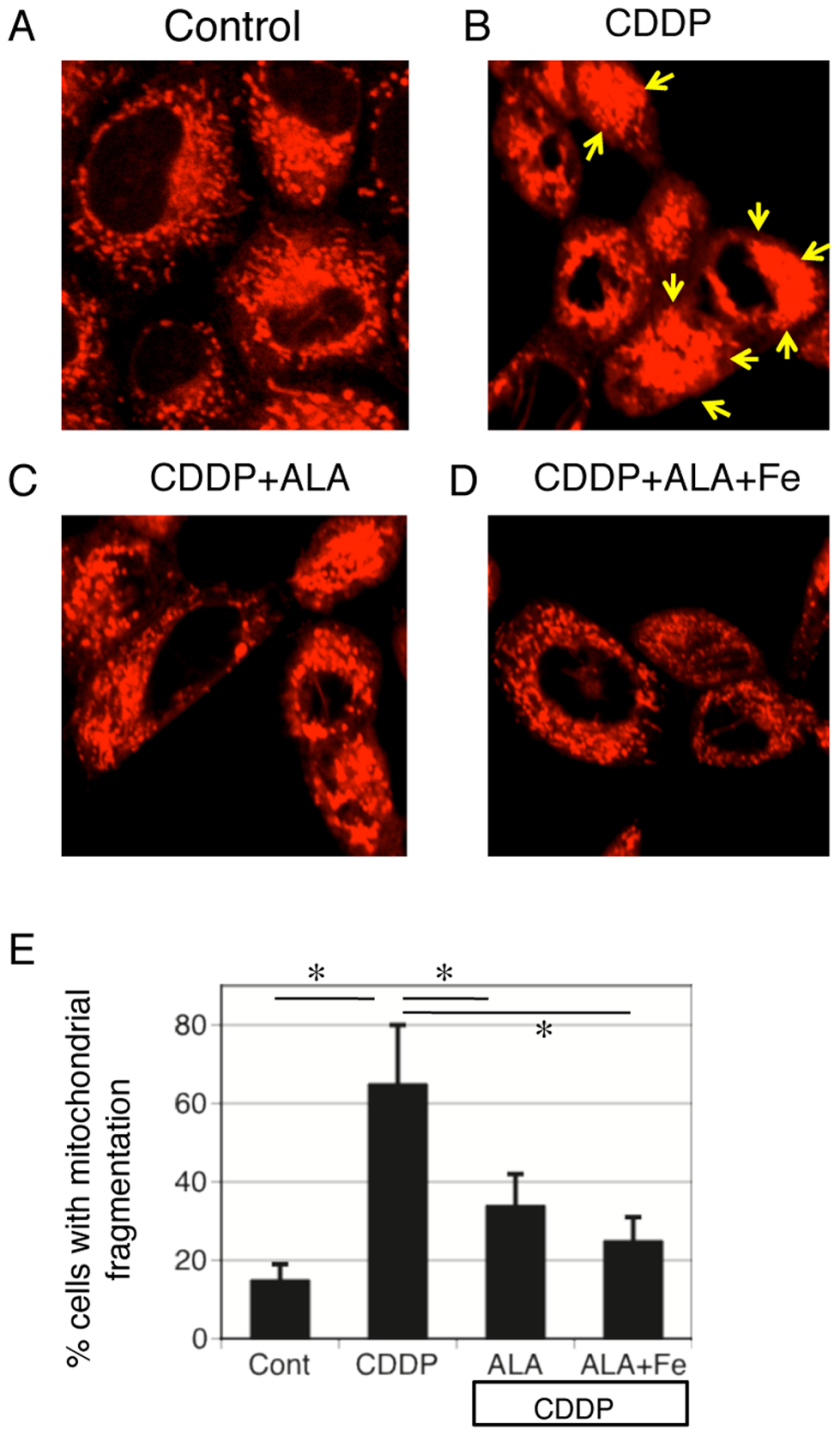
ALA and Fe prevent cisplatin-induced damage of mitochondrial structure in NRK-52E cells. (A–D) Confocal microscopy demonstrates mitochondria structure in NRK-52E cells transfected with mitochondria-targeted red fluorescent protein (mitoDsRed) vectors. NRK-52E cells were treated with in control condition, cisplatin, cisplatin + ALA, and cisplatin + ALA + Fe. Typical condensed rounded organelles in response to cisplatin were marked by arrows. (E) Quantitative analysis of the typical reticulotubular appearance of mitochondria in healthy NRK-52E cells and multiple rounded organelles are performed. ALA and Fe treatment prevented these structural changes in mitochondria. ALA only treatment partially prevented these structural changes in mitochondria. Bars represent the mean ± SEM, n = 6. *P<0.05 by ANOVA.

**Figure 9:**
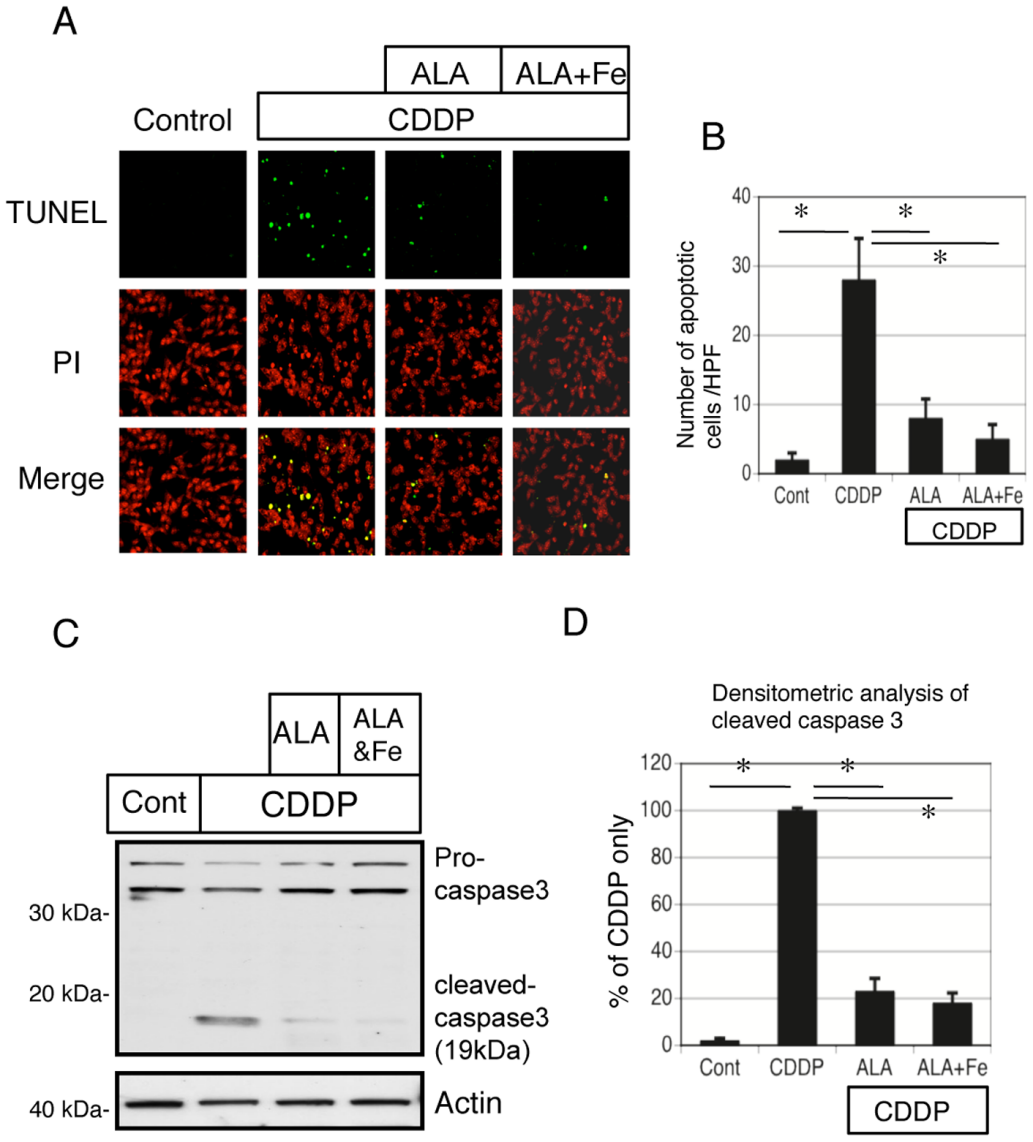
TUNEL assay and cleaved caspase 3 to evaluate apoptosis in NRK-52E cells exposed to cisplatin and ALA + Fe. (A) TUNEL assay to evaluate apoptosis (green) in NRK-52E cells exposed to cisplatin or control. Nuclei were stained with propidium iodide (PI) (red). TUNEL assay to evaluate apoptosis (green) in NRK-52E cells exposed to cisplatin + ALA or cisplatin + ALA + Fe. (B)The induction of the number of apoptotic cells by cisplatin was reduced by ALA and ALA + Fe using quantitative analysis. Data are presented as mean ± SEM, n = 6. *P<0.05 vs. pcDNA-transfected cells. (C) Western blot analysis of cleaved caspase3 were performed following incubation with in control condition, cisplatin, cisplatin + ALA, and cisplatin + ALA + Fe. (D) Quantitative densitometry was performed for cleaved caspase 3 blots. Data are presented as mean ± SEM, n = 6. *P<0.05 by ANOVA.

We examined the effects of ALA and Fe on mitochondrial structure by using a laser-scanning confocal microscope. The typical reticulotubular appearance of mitochondria in healthy NRK-52E cells (Figure 8A) had disintegrated into condensed rounded organelles in response to cisplatin at 12 h (arrows in Figure 8B). However, ALA and Fe treatment prevented these structural changes in mitochondria (Figure 8D,E). ALA-only treatment partially prevented these structural changes in mitochondria (Figure 8C,E). We also examined the effects of ALA and Fe in cisplatin-induced apoptosis in NRK-53E cells. We used TUNEL staining to evaluate apoptosis in NRK-52E cells, and found that cisplatin-induced apoptosis was significantly reduced by ALA and ALA + Fe (Figure 9A,B). These data are in accordance with the results of western blot analysis of cleaved caspase-3 (Figure 9C,D).

### Measurement of heme in ALA-treated, cisplatin-induced AKI rats and NRK-52E cells exposed to cisplatin and ALA + Fe

**Figure 10:**
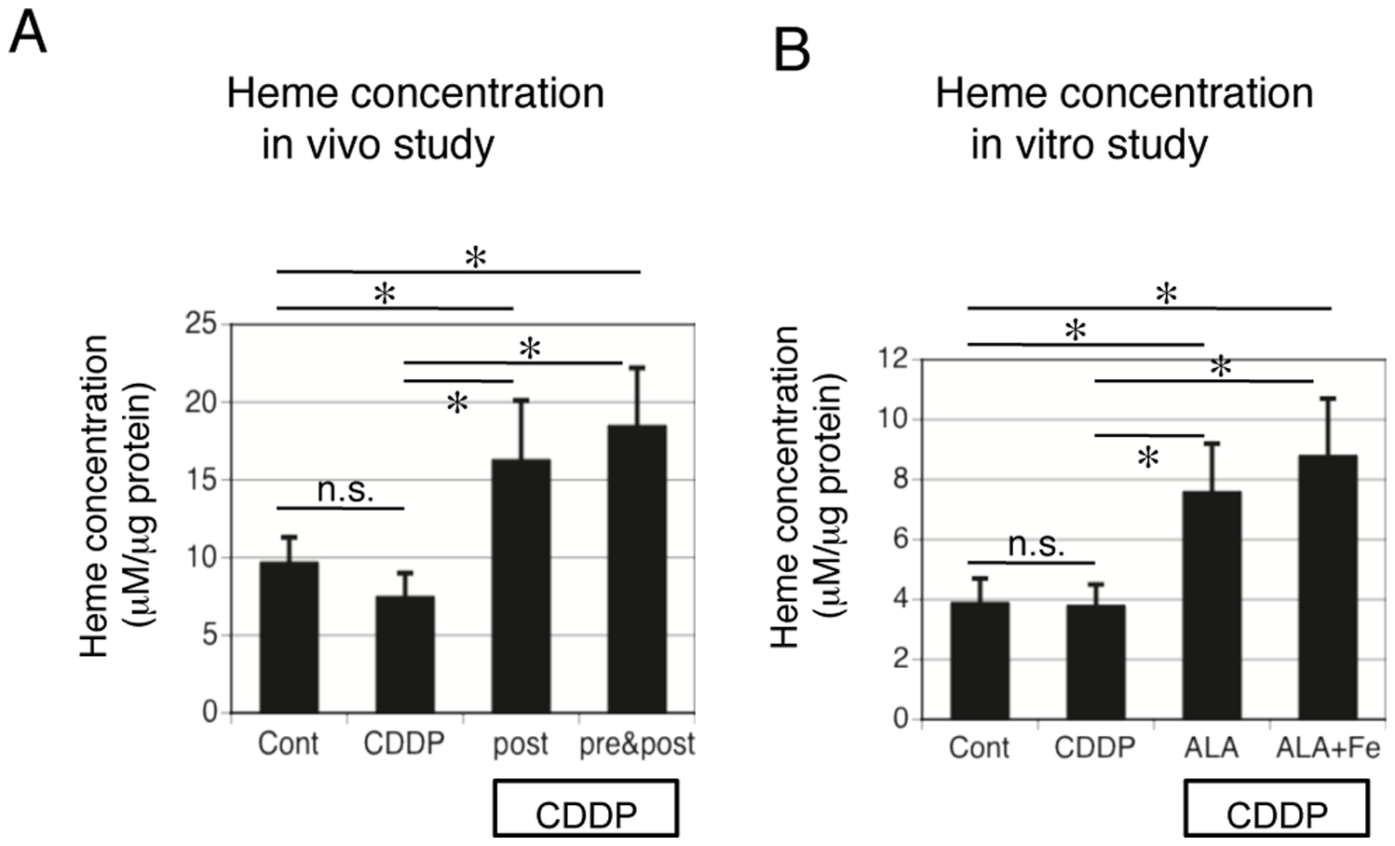
Measurement of heme in the ALA-treated cisplatin-induced AKI rats and NRK-52E cells exposed to cisplatin and ALA + Fe. (A) Aliquots (100 μg) of protein from renal tissue extracts were used for the heme assay in cisplatin-treated, control, and cisplatin + ALA (both post and pre + post)- treated rats. (B) Aliquots (10 μg) of protein extracts from NRK-52E cells were used for the heme assay in the control as well as the cisplatin-, cisplatin + ALA-, and cisplatin + ALA + Fe-treated NRK-52E cells. Data are the mean ± SEM of six experiments per group. *P<0.05 v.s. control or CDDP, n.s. is not significant by ANOVA.

We used a heme assay kit to evaluate the metabolic products of ALA. We examined the heme concentration in the renal tissues in the cisplatin-treated, control, and cisplatin + ALA-treated rats. As shown in Figure 10A, cisplatin did not significantly change the heme concentration. ALA treatment (both post and pre + post) significantly increased the heme concentration. We then examined whether ALA and Fe increased heme concentration in vitro. We measured the heme concentrations in the control, cisplatin-, cisplatin + ALA-, and cisplatin + ALA + Fe-treated NRK-52E cells. As shown in Figure 10, cisplatin did not change the heme concentration. ALA as well as ALA + Fe treatment significantly increased the heme concentration. These data demonstrated that heme is up-regulated by ALA treatment both in vitro and in vivo.

### Effects of 5-Aminolevulinic acid on the antitumorigenic effects of cisplatin in vivo

**Figure 11:**
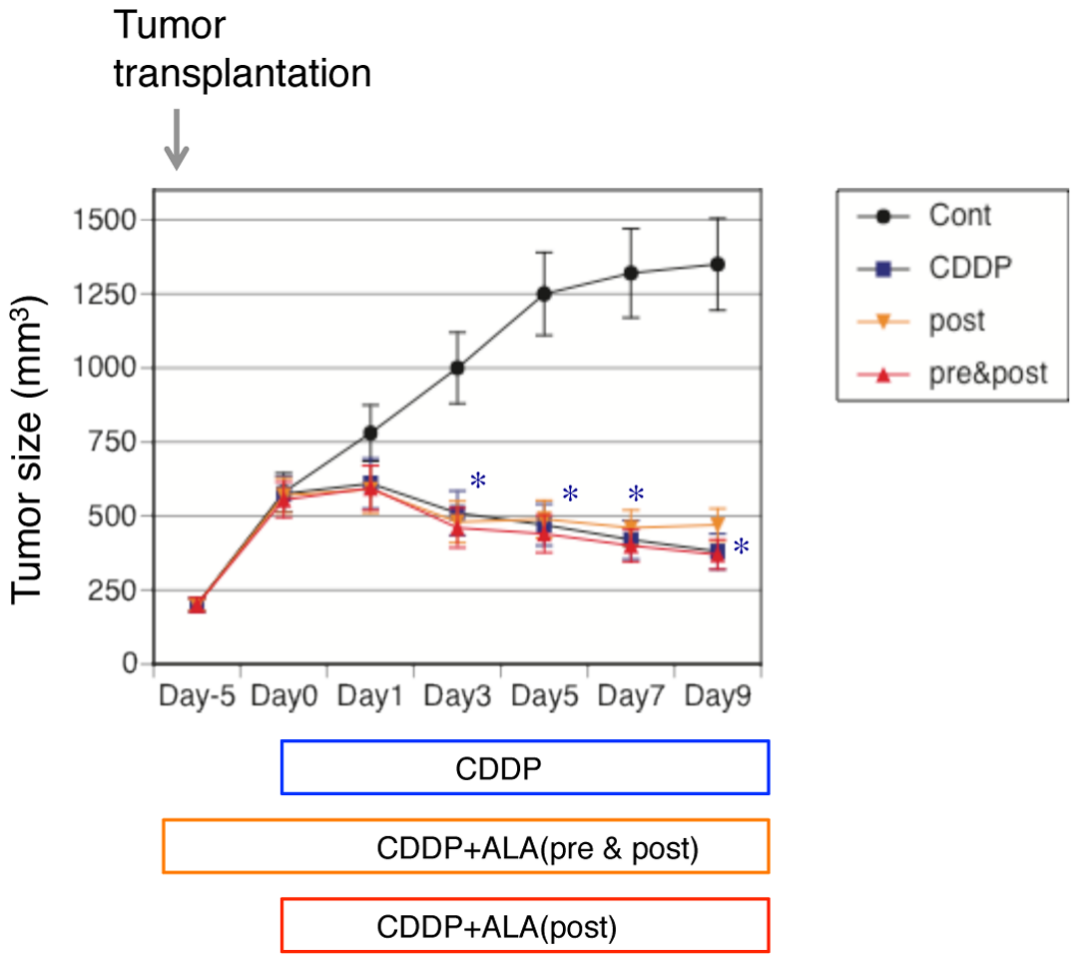
Effects of ALA on anti-tumorinogenic effects of cisplatin in vivo. We evaluated the size of transplantated bladder carcinoma to the F344/NJcl-rnu/rnu rats skin. Rats were divided into four subgroups: 1) a control (saline) group, 2) a cisplatin group, 3) an ALA–treated post cisplatin-injection group, 4) an ALA–treated pre & post cisplatin-injection group (n = 5 for each group), The diameter of the carcinoma were measured at 1, 3. 5, 7, and 9 days after surgery (n = 5/group). Cisplatin reduced size of transplantated renal carcinoma compared with control group. ALA (post and pre & post) did not change the anti cancer effects of cisplatin. Data are the mean ± SEM of 6 experiments per group. *P<0.05 v.s. control by ANOVA.

To evaluate if the ALA reduction of cisplatin nephrotoxicity was specific for the kidney, we looked for potential reductions in the chemotherapeutic efficacy of cisplatin in bladder carcinoma cells. We evaluated the size of the renal carcinoma transplanted into the rat skin, as shown in Figure 11, and found that cisplatin reduced the size. ALA (post and pre & post) did not change the anticancer effects of cisplatin.

## Discussion

In this study, we demonstrated that the protective role of ALA in cisplatin-induced AKI is through the protection of mitochondrial viability, and ALA prevents tubular apoptosis. In addition, ALA has no significant effects on the anticancer efficiency of cisplatin in rats. Thus, ALA has the potential to prevent cisplatin nephrotoxicity without compromising the anticancer efficacy of cisplatin. Cisplatin is a chemotherapeutic agent that is used in the treatment of a variety of solid-organ cancers, including those of the head, neck, testis, ovary, and breast [19]. Unfortunately, in addition to causing bone marrow suppression, ototoxicity, and anaphylaxis, around 30% of patients receiving cisplatin develop AKI owing to its preferential accumulation within the proximal tubule cells in the outer medulla of the kidney [20], [21]. The cellular events in cisplatin-mediated nephrotoxicity, including decreased protein synthesis, membrane peroxidation, mitochondrial dysfunction, and DNA injury, are a consequence of free radical generation and the body's inability to scavenge such molecules [22], [23]. Consistent with previous studies, we found that cisplatin-induced renal dysfunction and morphological changes were associated with mitochondrial injury in the rat kidney [9]. These findings were also confirmed by using NRK-52E cells, in which we documented that cisplatin caused mitochondrial fragmentation and decrease of mitochondrial enzymes.

In animal cells, ALA is formed from glycine and succinyl CoA by ALA synthase in mitochondria. COX and cytochrome *c* are hemoproteins. In this study, we demonstrated that COX-IV expression was increased by ALA administration in vivo and in vitro. This finding is in accordance with previous report with liver lysates [24]. ALA is the precursor of protoporphyrin, and heme is produced by the insertion of iron. Therefore, ALA administration can result in heme production in the rat kidney, and it is possible that COX-IV was increased by ALA administration. ALA administration upregulates HO-1 expression. HO-1 is a key enzyme for antioxidant response in renal tubular cells and therefore protects mitochondrial function and upregulates mitochondrial enzymes. We first demonstrated that typical mitochondrial enzymes, such as PGC-1α, UCP2, and ATP5α, were upregulated by ALA administration. Interestingly, ALA and Fe strongly induced these enzymes.

This is the first study to demonstrate that HO-1 is upregulated by ALA and Fe in vivo and in vitro. HO-1 a microsomal enzyme involved in the degradation of heme, resulting in the generation of biliverdin, iron, and carbon monoxide. Recent attention has focused on the biological effects of product(s) of this enzymatic reaction that have important antioxidant, anti-inflammatory, and cytoprotective functions [25]. Induction of HO-1 occurs as an adaptive and beneficial response to a wide variety of oxidant stimuli, including heme, hydrogen peroxide, cytokines, growth factors, heavy metals, nitric oxide, and oxidized LDL [25]. HO-1, the enzyme that is responsible for heme degradation, is upregulated in the proximal tubule cells in response to oxidant stress [26], and once induced, it confers dramatic cytoprotective and anti-inflammatory effects [25], [27]. The mechanisms of HO-1 regulation are reported by several pathways: one is hypoxia and inflammatory signals, including IL-1 and TNFα, and second are the nuclear factor E2-related factor-2 (Nrf2) and heme levels. HO-1 gene regulation is reported to involve Kelch-like enoyl-CoA hydratase (ECH)-associated protein 1 (Keap1) regulation through antioxidant response elements (ARE), Nrf2, and their binding in the cytosol [28]. Recently, investigators identified a heme-dependent degradation system involving iron regulatory protein 2 (IRP2) as a sensor of iron metabolism. IRP2 upregulates intracellular free iron and modulates intracellular iron stores, and increased iron efflux has been suggested as a mechanism for the cytoprotective effects of HO-1 expression [29]. We clearly demonstrated that cisplatin itself upregulates HO-1 expression; these findings were in accordance with those of the previous reports [30], [31]. Furthermore, we demonstrated that ALA induced HO-1 and ALA + Fe additively upregulated HO-1 mRNA and protein expression in NRK-52E cells. The additional induction of HO-1 by Fe may confer cytoprotective and antioxidant responses in renal tubular cells and protect mitochondrial function and enzymes such as PGC-1α, ATP5α, and UCP2. However, further studies are needed to completely clarify the mechanisms of heme regulation and their associated metabolic pathways concerning mitochondrial function.

No previous studies before ours have demonstrated the protective effects of ALA against cisplatin-induced apoptosis in vivo and in vitro. Cisplatin induces apoptosis of renal proximal tubule cells (LLC-PK1) in vitro through mitochondria-dependent and -independent pathways [3], partly through the activation of caspase-3 and oxidative stress [4], [5]. Several studies suggest that caspase inhibitors or knockout of apoptosis-related genes attenuate ischemia-induced AKI in rats [32]. In our experiments, cisplatin administration induced apoptosis in vivo and vitro, as confirmed by TUNEL staining and cleaved caspase-3 level. Our data clearly demonstrated that ALA inhibited cisplatin-induced apoptosis in vivo and vitro, which was evaluated by examining the results of TUNEL staining and cleaved caspase-3 levels. Furthermore, ALA + Fe additionally reduced cisplatin-induced apoptosis in NRK-52E cells. The plausible mechanisms of the ALA + Fe effects on antiapoptosis are antioxidative effects and protective effects toward mitochondria in renal tubular cells. In cancer cells, several studies suggested that ALA induces apoptosis through the accumulation of PpIX. ALA-mediated accumulation of PpIX causes photosensitization of cancer cells and is used in the treatment of hepatocellular carcinoma, oral cancer, and bladder carcinoma [13], [33]. In several cancer cell lines, ABCG2 transporter is highly expressed and PpIX is accumulated in the cytosol and causes cytotoxic damage and apoptosis [10]. The mechanisms of the differential effects of ALA in the apoptotic pathway in normal renal tubular cells and carcinoma cells are not well known and need to be studied in future.

Cisplatin is one of the most effective and potent anticancer drugs in the treatment of epithelial malignancies [1]. Considering the clinical use of ALA in preventing cisplatin-induced nephrotoxicity, it should be checked whether ALA interferes with the anticancer effects of cisplatin. Thus, we examined the effects of ALA on the size of the renal carcinoma transplanted into rat skin. As shown in Figure 10, cisplatin reduced the size of the transplanted renal carcinoma. ALA (post and pre & post) did not change the anticancer effects of cisplatin. At least in our experimental condition, ALA did not interfere with the antitumorigenic effects of cisplatin in vivo. Further research is needed to gain insight into the effects of ALA on the anticancer effects of cisplatin before clinical use.

In summary, our study has produced 2 novel findings. First, the protective role of ALA in cisplatin-induced AKI is through the protection of mitochondrial viability, induction of HO-1, and prevention of tubular apoptosis. Second, ALA has no significant effects on the anticancer efficiency of cisplatin in rats and prevents tubular apoptosis. Further studies are necessary to gain a more precise understanding of the molecular mechanisms by which ALA protects renal cells against cisplatin-induced nephrotoxicity.

## Supporting Information

Figure S1 **Experimental designs for in vivo study.** The rats were given a single intraperitoneal injection of either a vehicle (saline) or cisplatin (8 mg/kg body weight). 5-Aminolevulinic acid (ALA) 10 mg/kg + Fe (sodium ferrous citrate 15.7 mg/kg) were dissolved in drinking water (10 ml/kg) were administered. Rats were divided into four subgroups: 1) a control (saline) group, 2) a cisplatin group, 3) an ALA–treated post (0–9 days after CDDP injection) cisplatin-injection group, 4) an ALA–treated pre(5 days before CDDP injection) & post cisplatin-injection group (n = 8 for each group). Blood samples were obtained for measurement of blood urea nitrogen and serum creatinine. at 1, 3. 5, 7, and 9 days after CDDP injection. Rats were sacrificed at day 5 and 9, and renal tissue are obtained.
(TIF)
