# Supplementary figures and images for: 5-Aminolevulinic Acid Protects against Cisplatin-Induced Nephrotoxicity without Compromising the Anticancer Efficiency of Cisplatin in Rats In Vitro and In Vivo
Source: PLoS One. 2013 Dec 6;8(12):e80850. doi: 10.1371/journal.pone.0080850 (PMC3855642; doi:10.1371/journal.pone.0080850)

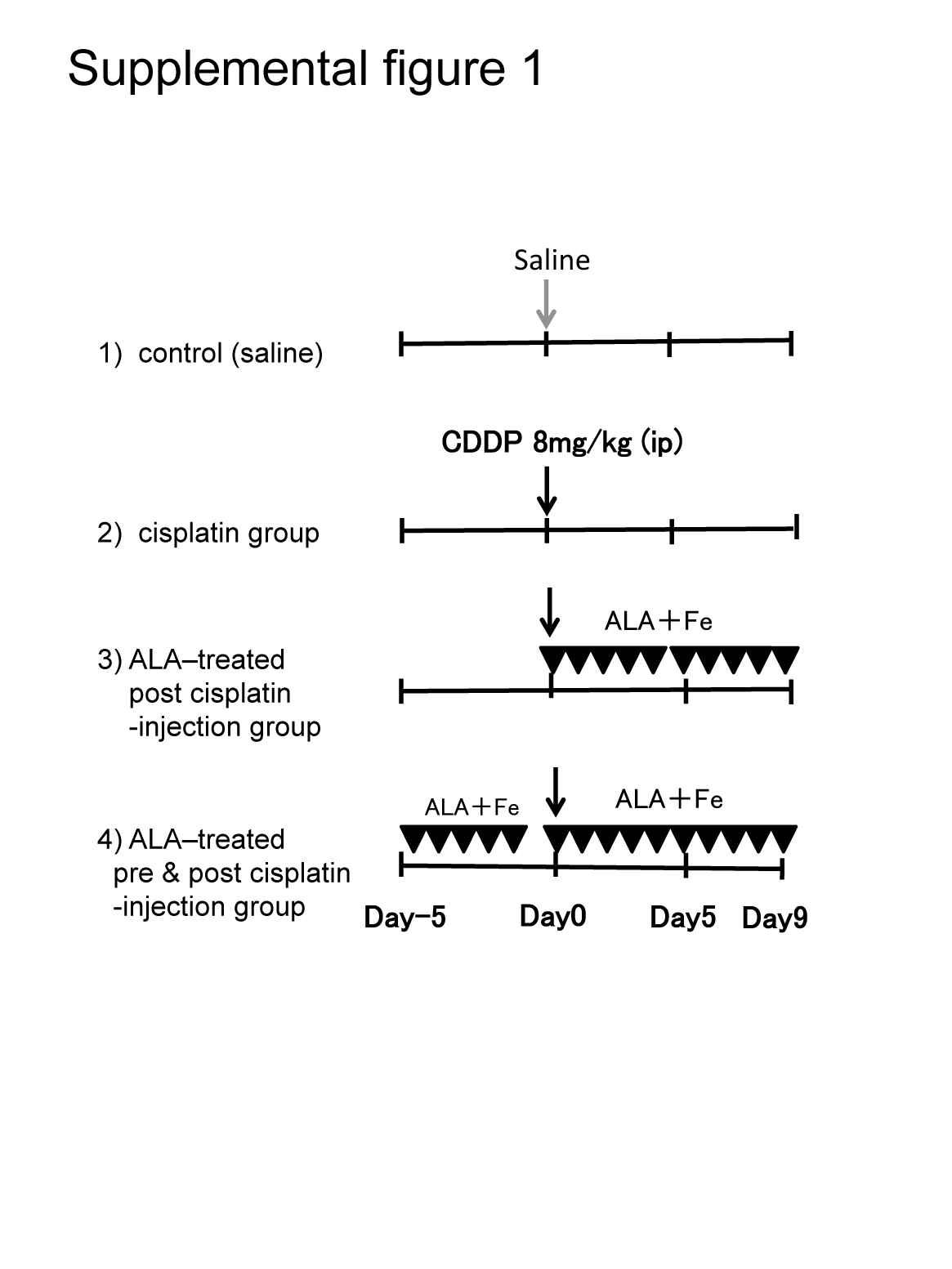

Supplement: Figure S1 — Experimental designs for in vivo study. The rats were given a single intraperitoneal injection of either a vehicle (saline) or cisplatin (8 mg/kg body weight). 5-Aminolevulinic acid (ALA) 10 mg/kg + Fe (sodium ferrous citrate 15.7 mg/kg) were dissolved in drinking water (10 ml/kg) were administered. Rats were divided into four subgroups: 1) a control (saline) group, 2) a cisplatin group, 3) an ALA–treated post (0–9 days after CDDP injection) cisplatin-injection group, 4) an ALA–treated pre(5 days before CDDP injection) & post cisplatin-injection group (n = 8 for each group). Blood samples were obtained for measurement of blood urea nitrogen and serum creatinine. at 1, 3. 5, 7, and 9 days after CDDP injection. Rats were sacrificed at day 5 and 9, and renal tissue are obtained. (TIF) [file pone.0080850.s001.tif]
